# Gene Variants Implicated in Steatotic Liver Disease: Opportunities for Diagnostics and Therapeutics

**DOI:** 10.3390/biomedicines11102809

**Published:** 2023-10-17

**Authors:** Gary Huang, Daniel F. Wallace, Elizabeth E. Powell, Tony Rahman, Paul J. Clark, V. Nathan Subramaniam

**Affiliations:** 1 Hepatogenomics Research Group, Queensland University of Technology (QUT), Brisbane, QLD 4059, Australia; gary.huang@hdr.qut.edu.au; 2 Centre for Genomics and Personalised Health, Queensland University of Technology (QUT), Brisbane, QLD 4059, Australia; d5.wallace@qut.edu.au; 3 School of Biomedical Sciences, Queensland University of Technology (QUT), Brisbane, QLD 4059, Australia; 4 Metallogenomics Laboratory, Queensland University of Technology (QUT), Brisbane, QLD 4059, Australia; 5 QIMR Berghofer Medical Research Institute, Brisbane, QLD 4006, Australia; Elizabeth.Powell@qimrberghofer.edu.au; 6 Department of Gastroenterology and Hepatology, Princess Alexandra Hospital, Brisbane, QLD 4102, Australia; 7 Centre for Liver Disease Research, Translational Research Institute, Faculty of Medicine, The University of Queensland, Brisbane, QLD 4101, Australia; 8 Department of Gastroenterology and Hepatology, Prince Charles Hospital, Brisbane, QLD 4032, Australia; tonyrahman@gmail.com; 9 Mater Adult Hospital, Faculty of Medicine, The University of Queensland, Brisbane, QLD 4101, Australia; paul.j.clark@uq.edu.au

**Keywords:** NAFLD, NASH, MASLD, MASH, steatotic liver disease, lipid metabolism, genetics, variants

## Abstract

Non-alcoholic fatty liver disease (NAFLD) describes a steatotic (or fatty) liver occurring as a consequence of a combination of metabolic, environmental, and genetic factors, in the absence of significant alcohol consumption and other liver diseases. NAFLD is a spectrum of conditions. Steatosis in the absence of inflammation is relatively benign, but the disease can progress into more severe forms like non-alcoholic steatohepatitis (NASH), liver cirrhosis, and hepatocellular carcinoma. NAFLD onset and progression are complex, as it is affected by many risk factors. The interaction between genetic predisposition and other factors partially explains the large variability of NAFLD phenotype and natural history. Numerous genes and variants have been identified through large-scale genome-wide association studies (GWAS) that are associated with NAFLD and one or more subtypes of the disease. Among them, the largest effect size and most consistent association have been patatin-like phospholipase domain-containing protein 3 (*PNPLA3*), transmembrane 6 superfamily member 2 (*TM6SF2*), and membrane-bound O-acyltransferase domain containing 7 (*MBOAT7*) genes. Extensive in vitro and in vivo studies have been conducted on these variants to validate these associations. The focus of this review is to highlight the genetics underpinning the molecular mechanisms driving the onset and progression of NAFLD and how they could potentially be used to improve genetic-based diagnostic testing of the disease and develop personalized, targeted therapeutics.

## 1. Introduction

### 1.1. The Liver and Steatotic Liver Disease

The liver is a vital metabolic organ that is responsible for the co-ordination and regulation of many endocrine and exocrine functions [1]. These functions are important for alcohol and drug detoxification [2,3], bile production [4], fat-soluble vitamin storage [5], and energy and lipid metabolism [6,7]. The liver’s key role in homeostasis translates to high patient morbidity and mortality rates within the development of liver disease [1].

Non-alcoholic fatty liver disease (NAFLD) describes the deposition of excessive fats in the liver in the absence of significant alcohol consumption and other liver diseases (such as hemochromatosis, hepatitis B and C, and Wilson disease) [8,9,10]. Recently, the nomenclature describing NAFLD (and the non-alcoholic steatohepatitis (NASH) subtype) has been revised, as the existing language does not fully reflect the aetiology of the disease (non-alcoholic) and is stigmatising (fatty) [11,12]. The term metabolic dysfunction associated fatty liver disease (MAFLD) nomenclature was proposed in 2020 [12,13]. MAFLD is inclusive of alcohol intake because it recognises that NAFLD and alcohol-related liver disease (ALD) aetiologies share some underlying molecular mechanisms and pathophysiological processes [12]. However, there is still contention with the mixing of aetiologies and social stigma with using the term “fatty” [11,14]. In a 2023 response, the NAFLD Nomenclature Initiative was announced [11,14]. This initiative was a collaboration between the American Association for the Study of Liver Diseases (AASLD), the Asociación Latino-Americana para el Estudio del Hígado (ALEH), the European Association for Study of the Liver (EASL), and other global academic professionals. The initiative proposed using metabolic dysfunction-associated steatotic liver disease (MASLD) to replace NAFLD, metabolic dysfunction-associated steatohepatitis (MASH) to replace NASH, and MetALD (metabolic alcohol-related liver disease) to encompass MASLD patients with significant alcohol intake [11]. The term “metabolic” is more inclusive as it recognizes cardiometabolic factors such as obesity and type 2 diabetes mellitus as risk factors involved in the underlying pathophysiology, rather than classifying according to alcohol intake (i.e., alcoholic and non-alcoholic) [11,13]. Individuals with a steatotic liver with no associated cardiometabolic factors or known cause are considered to have cryptogenic steatotic liver disease (SLD) [11]. As these changes in nomenclature of steatotic liver disease are still to come into wide use, in this article they will continue to be referred to as NAFLD (and NASH).

**Figure 1:**
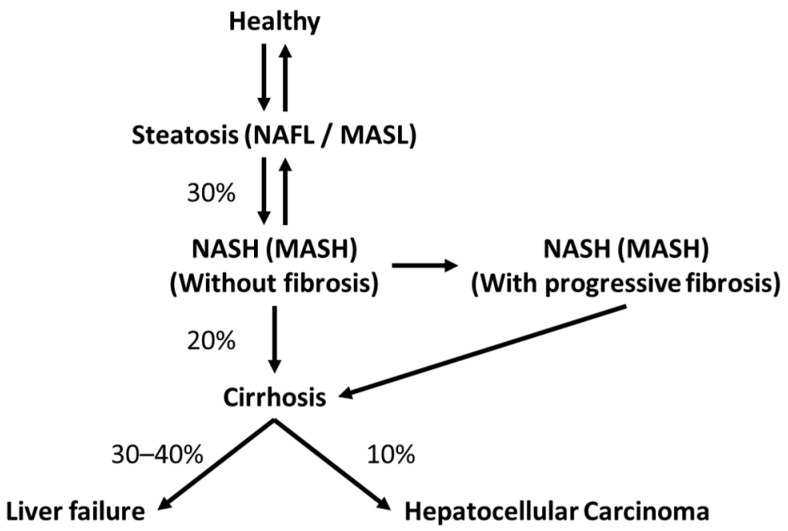
NAFLD progression. Damage from liver steatosis, NASH and early fibrosis are potentially reversible with treatment. However, damage resulting in cirrhosis is not typically reversible [15]. This figure was adapted to include new NAFLD nomenclature.

It is estimated that up to a quarter of the global adult population is afflicted with NAFLD [10]. NAFLD encompasses a spectrum of conditions with varying degrees of impact. As seen in Figure 1, these conditions range from hepatic steatosis alone with no or little necroinflammation to steatohepatitis (lipid accumulation with inflammation and liver cell injury (ballooning)) with or without fibrosis, and cirrhosis [8,9,10]. It is estimated that approximately 30% of NAFLD patients will develop NASH and about one-fifth of those with NASH will progress directly to cirrhosis [15]. Roughly 30–40% of patients with cirrhosis will decompensate, leading to liver failure and death over 10 years, whereas up to 10% of patients with cirrhosis may develop hepatocellular carcinoma (HCC) [15,16].

### 1.2. NAFLD Pathogenesis

The acquisition of and phenotype of NAFLD is complex, as it involves interactions between the environment and host genetics [8,9]. However, a few critical processes are recognised in the disease, such as the accumulation of fat in hepatocytes, subsequent cell injury and oxidative stress with inflammation, and apoptotic cell death [17]. Two theories (“two-hit” and “multiple-hit” hypotheses) have been proposed to describe the progression of NAFLD to NASH [18,19].

The two-hit hypothesis is the earliest explanation for NAFLD progression. It was proposed that lipid accumulation in hepatocytes was the trigger (first hit) for NAFLD and was intensified by increased fat intake and insulin resistance, which can be triggered by poor nutritional habits and a sedentary lifestyle [18,19]. This condition would prime the liver to be more susceptible to the second hit, which was the main driving force of NAFLD to NASH progression. Second-hit factors include damage from inflammation and oxidative stress by overproduced reactive oxygen species (ROS), metabolic dysfunction, and genetic polymorphisms that increase the risk for the disease [18,19,20,21,22]. 

**Figure 2:**
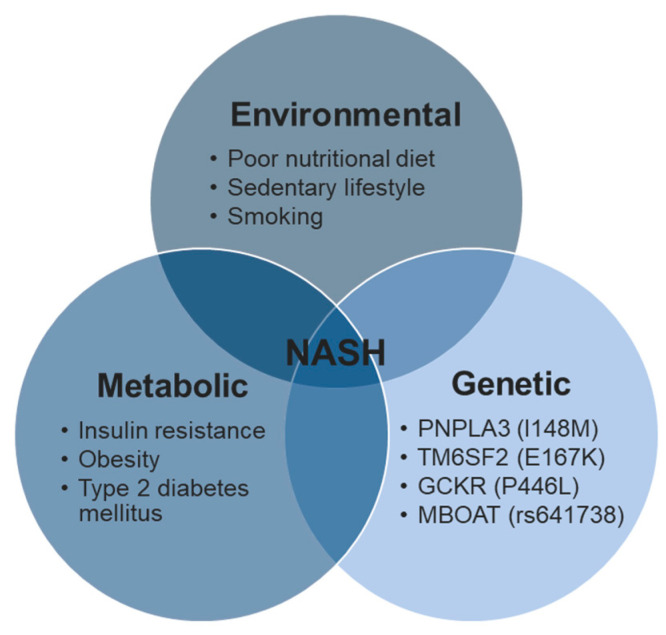
Examples of risk factors contributing to the promotion of NASH onset according to the multiple-hit hypothesis. Risk factors for NASH can be categorised into environmental, metabolic, and genetic, and their interactions with each other are presented in a Venn diagram.

Currently, the popular opinion on NAFLD progression is the multiple hit theory, which describes multiple concurrently occurring insults and stressors (environmental and metabolic) interacting together to induce NAFLD in genetically predisposed individuals (Figure 2) [20,21,22,23]. For instance, individuals genetically predisposed to metabolic syndromes may acquire insulin resistance from poor nutritional habits and a sedentary lifestyle [19]. Insulin resistance in adipose tissues increases free fatty acid (FFA) flow to the liver and promotes lipotoxicity and oxidative stress. At the same time, the adipose tissue may release inflammatory cytokines (adipokines), which coupled with oxidative stress to promote chronic liver inflammation, cell death, and activation of hepatic stellate cells that promote fibrosis [18,19].

In a healthy liver, triglyceride content is normally maintained at about 5% of liver weight via tightly regulated mechanisms in lipid metabolism [7,20,24,25]. However, an imbalance between fatty acid (FA) availability (increased FA synthesis and uptake) and FA removal (decreased β-oxidation) can induce hepatic FA and triglyceride overload, both hallmarks of NAFLD [20,26]. Increased fat content may be associated with liver damage due to increased production of reactive oxygen species and promotion of inflammation [20].

### 1.3. Lipid Metabolism

#### 1.3.1. Fatty Acid Synthesis and Uptake

**Figure 3:**
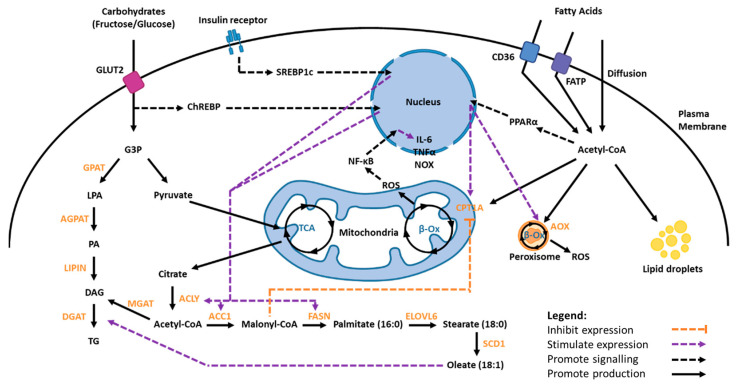
Overview of lipid metabolism. Dietary carbohydrates and lipids are major contributors of fatty acids. Carbohydrates may be converted into triglycerides via the G3P pathway or fatty acids via DNL through the mitochondrial tricarboxylic acid cycle. The latter is regulated by both the ChREBP and SREBP1c signalling pathways. Fatty acids can be stored in cytoplasmic lipid droplets until required or be oxidised for removal. β-oxidation of lipids can occur in the mitochondria or peroxisomes and is mainly regulated by the PPARα signalling pathway. This figure was created with BioRender.com.

In the liver, FAs can be derived from the diet or from three major endogenous sources: de novo lipogenesis (DNL), plasma-derived non-esterified FAs from adipose tissue, and cytoplasmic lipid droplets (Figure 3) [7,27]. After a meal (fed state), dietary lipids in the intestinal lumen are emulsified by bile salts and hydrolysed by pancreatic lipase [4,7]. This produces FFAs, which are then re-synthesised into triglycerides (the most common non-toxic form of FA) in enterocytes before being packaged into triglyceride-rich lipoproteins known as chylomicrons [7,27]. 

DNL is the mechanism by which FAs are converted from glucose when there are high levels of carbohydrates. This mechanism is transcriptionally regulated by plasma glucose, glucagon, and insulin levels [7,27,28]. When fasting (fasted state), plasma insulin levels are low. This increases lipolytic activity in adipose tissues, promoting the release of non-esterified FAs into blood, thereby increasing the available plasma FA pool for the liver to uptake [7]. Most FAs in the circulation are bound to albumin but can enter hepatocytes via the FA transport protein (FATP), FA translocase (FAT)/cluster of differentiation 36 (CD36), or diffusion through the lipid bilayer [7]. Inside the hepatocytes, acyl-CoA synthetase (ACS) and FA-binding protein (FABP) bind to the long-chain FAs and transport them to intracellular compartments for downstream metabolic processes, such as the esterification into glycerol-3-phosphate (G3P) and cholesterol, or to the nucleus for transcription modulation [7,27]. 

Over 90% of total triglyceride synthesis is processed through the G3P pathway, which enzymatically converts G3P into triglycerides and safely stores large amounts of FAs in cells [7]. These enzymes include G3P acyltransferase (GPAT), lysophosphatidic acid acyltransferase (LPAAT), monoacylglycerol acyltransferase (MGAT), and diacylglycerol acyltransferase (DGAT) [7,29,30]. Studies have shown that the overexpression of SREBP1c in adipocytes and the liver positively regulates mitochondrial GPAT expression [31,32].

#### 1.3.2. Fatty Acid Oxidation

Fatty acid oxidation is the process in which FAs are catabolised for energy [27]. This can occur in the mitochondria or peroxisomes (Figure 3). PPARα acts as a sensor for FAs, so impaired expression and function can result in steatosis. Apart from PPARα, other regulatory genes involved in β-oxidation include *ACC2*, long-chain acyl-CoA synthetase 1 (*ACSL1*) and carnitine palmitoyltransferase 1 (*CPT1*) in the mitochondria, as well as acyl-CoA oxidase (*AOX*) in peroxisomes and *cytochrome P450 4A* in the endoplasmic reticulum [7,27].

CPT1 is the key rate-limiting enzyme involved in the transfer of FAs into the mitochondria from the cytosol [33]. PPARα and its co-activator PPAR gamma co-activator 1α (PGC-1α) are involved in enhancing CPT1 expression, as well as ACOX expression. ACSL1 is a PPARα target that interacts with CPT1 through the shuttling of acyl-CoA at the outer mitochondrial membrane [7]. It has been suggested that ACSL1 is responsible for maintaining substrate availability for β-oxidation. However, this process is yet to be fully understood due to conflicting data [7,34]. 

Farnesoid X receptor (FXR) is a highly expressed liver receptor (particularly in endothelial, hepatocytes, and Kupffer cells) that has been shown, when activated, to reduce lipogenesis, inflammation, and liver fibrosis in individuals with NAFLD, NASH, or fibrosis and mice fed with a high-fat and high-carbohydrate diet [35,36,37]. This is achieved by inhibiting *SREBP1c* transcription, which reduces the amount of available FFAs, downregulating adipocyte-derived tumour necrosis factor α (*TNFα*) and increasing *PPARα* and *CPT1* expressions [38].

### 1.4. NAFLD Risk Factors: Genetics

There are numerous risk factors for developing NAFLD, which can be categorised as environmental, metabolic, and genetic [23]. As the focus of this review is the genetics of NAFLD, the readers are encouraged to considers these publications that have dealt with the environmental and metabolic risk factors associated with NAFLD [23,26,39,40,41,42,43,44,45,46,47,48].

There is no doubt that cultural and socioeconomic factors have a role in NAFLD acquisition and progression. However, the interaction between genetic risk factors and the environment is also heavily implicated in explaining the observed variability of NAFLD phenotype and severity among individuals. According to a 2016 meta-analysis, certain ethnic populations are more prone to acquiring NAFLD [10]. The Middle East (31.8%) and South America (30.5%) are reported to have the highest prevalence of NAFLD, as opposed to Africa (13.5%), which has the lowest. The prevalence rate of NAFLD in Europe and North America is approximately 24% [10]. This pattern of distribution for NAFLD is also seen on a national scale in the United States of America (USA) [49]. According to a more recent 2018 meta-analysis, the relative risk of NAFLD is highest and lowest in the Hispanic (RR = 1.47) and African-American (RR = 0.74) populations, respectively, compared to non-Hispanic Caucasians [49]. Even in high-risk cohorts (diabetic or obese), the African-American population was found to have a lower relative risk for NAFLD than non-Hispanic Caucasians (RR = 0.85).

### 1.5. NAFLD Heritability

Studies have shown that NAFLD and NASH have a strong heritability component based on common familial clustering [50,51]. In a 2001 study, nearly 18% of NASH patients from nine families had a first-degree relative (parent–child or sibling–sibling) who was similarly affected or worse [51]. Further evidence of NAFLD heritability was also presented in a 2009 study [52]. The prevalence of a fatty liver was found to be more common in siblings (59%) and parents of children with NAFLD (78%) compared to those who were overweight or obese but did not have NAFLD (17% and 37%, respectively) after adjusting for age, BMI, ethnicity, and sex [52].

Twin studies also support heritability in NAFLD [53,54]. A 2001 Danish-based twin study revealed that variations in liver function biomarkers (alanine aminotransferase (ALT), gamma-glutamyltransferase (GGT), lactate dehydrogenase (LDH), and bilirubin) were substantially impacted by genetic factors, and that these markers also had significant heritability (35–61%) [53]. Heritability did not change after adjusting for BMI and alcohol consumption. A 2009 Finnish-based twin study also showed that about 55% of variation in ALT and about 61% of variation in fasting serum insulin were attributed to heritability after adjusting for BMI and sex [54].

It has been suspected in recent years that there is missing heritability in NAFLD, which has prevented the testing of well-characterised variants to be implemented clinically for patient NAFLD diagnosis [55]. This missing heritability could be attributed to rare genetic variants. 

Rare gene variants having a minor allele frequency (MAF) of less than 1% are thought to play a unique role in disease genetics [56,57]. Rare variants have lower linkage disequilibrium with flanking variants, suggesting that disease associations are less likely due to non-random allele associations [56]. As a result, these variants may have a higher impact on gene expression and function, as well as high population specificity. However, there is a paucity of information on rare gene variants in NAFLD patients. This is due to the absence of functional genomics in many existing studies, which have contributed to the limited understanding about how rare gene variants affect liver function [55,58].

### 1.6. Gene Variants in NAFLD

Many liver disease-related gene variants have been identified from large-scale genome-wide association studies (GWAS) [59,60] or exome-wide association studies [61,62], which associate gene variants with diseases in patients. These studies have greatly contributed to the understanding of NAFLD genetics, pathogenesis, and variable prognosis [9]. Variants in the patatin-like phospholipase domain-containing protein 3 (*PNPLA3*) and transmembrane 6 superfamily member 2 (*TM6SF2*) genes are examples that have been suggested to contribute to the observed differences in NAFLD [63,64,65,66].

#### 1.6.1. PNPLA3

PNPLA3, or adiponutrin (ADPN), is a membrane-bound protein highly expressed in the liver and adipose tissue. It is responsible for non-specific hydrolase activity against various lipid substrates and is regulated by retinol levels [67,68]. In a 2008 genome-wide association study, Romeo and colleagues found that the change at codon 148 from isoleucine to methionine (p.I148M) (rs738409) was strongly associated with increased hepatic fat content [65]. Individuals carrying this variant had two-fold greater hepatic fat content compared to non-carriers. The *PNPLA3* p.I148M variant has since been well characterised and has been proposed to be a strong modifier of NAFLD pathogenesis [69]. Studies have suggested that this could be due to the variant inducing a gain- or loss-of-function to the PNPLA3 protein [68,70,71]. However, more recently, it has been discovered that overexpressing this variant impaired the PPARγ, retinoid X receptor, and retinoic acid receptor signalling pathways, while the Jun N-terminal kinase (JNK) and activator protein-1 signalling pathways were upregulated [72]. The dysregulation of these pathways increases inflammatory activity, which could explain why patients possessing the *PNPLA3* p.I148M variant are more susceptible to liver inflammation and steatohepatitis development [72]. In an even more recent study, it has been suggested that the *PNPLA3* p.I148M variant could instead be a neomorphic variant, in which the protein gains an altered (novel) function that is different from normal [73]. In the study by Romeo et al., the frequency of the *PNPLA3* p.I148M variant was determined to be highest in Hispanics (49%), followed by European-Americans (23%) and African-Americans (17%) [65]. Similar findings were also determined by Wagenknecht and colleagues who had used a different US cohort [66]. In a 2011 meta-analysis, the *PNPLA3* p.I148M variant was also associated with more than a three-fold greater risk for fibrosis and NASH compared to non-carriers [74]. Serum ALT levels were also measured in this study and were found to be 28% higher in those with the homozygous risk variant.

#### 1.6.2. TM6SF2

TM6SF2 is another well-known regulator of NAFLD through its positive association with plasma triglyceride levels [64]. In a 2014 study, the *TM6SF2* variant change at codon 167 from glutamic acid to lysine (p.E167K) (rs58542926) was found to be associated with approximately three-fold higher hepatic triglyceride in non-Hispanic European-Americans carrying the homozygous KK mutation compared to EE mutation carriers [63]. Further functional studies in this paper using *Tm6sf2* short hairpin ribonucleic acid (shRNA) knockdown mice also supported this finding [63]. However, the exact function of TM6SF2 remains unknown. In a 2016 multiethnic study, homozygotes for the *TM6SF2* p.E167K variant were found to be more prevalent in the Hispanic population (MAF = 0.089) than Caucasian (MAF = 0.061) or African-American (MAF = 0.033) populations [75]. This study also discovered that Caucasians and Hispanics homozygous for the variant had significantly elevated ALT and aspartate aminotransferase (AST) levels compared to heterozygous carriers. The African-American and Caucasian populations were also found to have significantly higher fat content in the liver, while Hispanics did not when comparing between homozygous and heterozygous carriers [75].

#### 1.6.3. MBOAT7

Membrane-bound O-acyltransferase domain containing 7 (MBOAT7) is involved in phospholipid remodelling via the incorporation of unsaturated fatty acids into lyso-phospholipids [76]. Carriers of the common rs641738 *MBOAT7* variant (missense change about 500 bp upstream of the gene) had increased hepatic fat content, risk for fibrosis and severe liver damage compared to individuals without the variant [77]. In fact, this variant may be associated with the full spectrum of NAFLD including HCC [78]. This is attributed to the variant reducing *MBOAT7* gene expression in the liver [76,77], which therefore reduces the amount of arachidonic acid being bound to lyso-phosphatidylinositol [76]. The rs641738 *MBOAT7* variant is most common in the European-American population (MAF = 0.42), followed by the African-American (MAF = 0.34) and Hispanic (MAF = 0.33) populations [76]. A recent 2021 study showed that *Mboat7* knockout mice fed with a high fat, methionine-low, and choline-deficient diet had significantly higher hepatic triglyceride levels than those fed with a normal diet [79]. There was also increased fibrotic development in the knockout mice with minimal impact on inflammation. These changes were also observed in biopsy-proven NAFLD individuals with the rs641738 *MBOAT7* variant, which suggested that hepatic fibrosis development occurred independently from inflammation. Additional findings highlighted similarities between Mboat7 knockout mice and NAFLD individuals with the homozygous *MBOAT7* variant; similar changes in lipid composition and species between these models were observed [79]. 

#### 1.6.4. GCKR

Glucokinase regulator (GCKR) is a liver glucokinase inhibitor involved in glucose metabolism [80,81]. Individuals who carried either the rs780094 (intronic variant) or the rs1260326 (change at codon 446 from proline to leucine (p.P446L)) *GCKR* variants displayed similar metabolic trait changes that were both associated with NAFLD and NASH [81]. Both variants are in strong linkage disequilibrium with each other [81,82]. The *GCKR* p.P446L variant in particular is more well known and is responsible for inducing loss-of-function of the gene [76], which interferes with the fructose-6-phosphate negative feedback inhibition mechanism on glucokinase [78]. This increases glucose uptake and metabolism, the availability of malonyl-CoA for increased DNL, and inhibition of CPT1A for reduced β-oxidation [80]. The *GCKR* p.P446L variant is more prevalent in the African-American population (MAF = 0.86) compared to the Hispanic (MAF = 0.67) and European-American (MAF = 0.60) populations [76].

#### 1.6.5. HSD17B13

Hydroxysteroid 17-beta dehydrogenase 13 (HSD17B13) is a hepatocyte-specific and lipid droplet-associated protein [83,84]. *HSD17B13* gene expression is about six-fold higher in patients with NASH than healthy controls (*p* = 0.003) [84]. Similar to TM6SF2, not much is known about the exact function of the *HSD17B13* gene [85]. However, members of the HSD17B family are responsible for promoting NAD(P)H/NAD(P)+-dependent oxidoreductase activity, which in turn modulates the balance between the less potent 17-ketosteroids and more potent 17β-hydroxysteroids [83]. The rs72613567 variant is one of the most common *HSD17B13* variants identified in patients with NAFLD (MAF = 0.194) and has high linkage disequilibrium with the *HSD17B13* rs6834314 variant [84]. The rs72613567 variant introduces an additional (duplicate) adenine nucleotide, which results in loss-of-function due to poor enzymatic activity [83,84]. Studies have shown that the rs72613567 variant is associated with decreased risk for ALD, NASH, and chronic liver disease [84,85,86]. This was due to the variant alleviating the severity of liver damage from inflammation and fibrosis, which was evident by reduced serum ALT and AST levels. These results highlight a protective role of the rs72613567 variant, which is to reduce the risk of further disease progression from steatosis [84,85,86]. In conjunction with *PNPLA3*, *TM6SF2,* or *MBOAT7* variants, the *HSD17B13* rs72613567 variant has been used to improve the accuracy for predicting NASH severity and advanced fibrosis [83]. There are several other *HSD17B13* variants located in non-coding genomic regions that have been associated with liver fat content and liver enzyme concentrations [84].

#### 1.6.6. Other Gene Variants

Recent GWAS studies have highlighted numerous new gene variants that may be associated with NAFLD pathogenesis in their respective populations [60,87,88]. The genes associated with these variants include apolipoprotein E (*APOE*) (rs429358), glycerol-3-phosphate acyltransferase mitochondrial (*GPAM* or *GPAT*) (rs2792751), interleukin 17 receptor A (*IL17RA*) (rs5748926), mitochondrial amidoxime reducing component 1 (*MARC1* or *MTARC1*) (rs2642438 and rs2642442), tribbles pseudokinase 1 (*TRIB1*) (rs2954021), and one locus near zinc finger protein 90-cadherin 1 (*ZFP90-CDH1*) (rs698718). Most of these genes are associated with the mitochondria, lipoprotein signalling or inflammation. However, only a small number of variants have been characterised through functional studies.

APOE is involved in synthesis of high-density and very-low-density lipoproteins, as it encodes for one of the protein components [89]. APOE is suspected to be involved in lipid metabolism because plasma expression is elevated in individuals with NAFLD [90]. However, the underlying mechanisms linking APOE expression and NAFLD are not well understood [87]. The 5’ adenosine monophosphate-activated protein kinase (AMPK)/mammalian target of rapamycin (mTOR) autophagy axis could explain this knowledge gap [89]. In a 2020 study, *ApoE* knockout mice fed a high-fat diet exhibited more severe inflammation, fibrosis, and steatosis than wild-type mice [89]. It was suspected that ApoE deficiency caused mitochondrial dysregulation, which reduced AMPK/mTOR. This, in turn, increased oxidative stress (elevation of ROS) and inflammation but reduced autophagy, which therefore prolonged liver damage.

The *GPAM* gene encodes a mitochondrial enzyme that is implicated in the first step of G3P-mediated triglyceride synthesis [88]. It promotes lipid accumulation by shifting lipids away from oxidation [91]. *GPAM* gene expression was found to be increased by about five-fold in mice with steatosis or NASH with fibrosis, developed through being fed a fast-food diet [91]. On the contrary, *Gpam* knockout mice were found to promote lipid oxidation as GPAM no longer competes with CPT1A for acyl-CoAs, which reduced the susceptibility of mice to oxidative stress [91]. The rs2792751 *GPAM* p.I43V variant is associated with increased cholesterol levels and risk for NAFLD [88].

The *IL17RA* gene encodes a cytokine that is involved in immunity [60,92]. Liver expression of IL17RA is higher with obesity and is associated with increased inflammation and liver damage [60,92]. In contrast, reduced protein synthesis has been shown to protect against high-fat-induced stress leading up to NASH [92].

Similar to *GPAM*, *MARC1* also encodes a mitochondrial enzyme, although the mechanisms underlying the association between MARC1 expression with NAFLD are not well understood [88]. Missense *MARC1* variants are associated with reduced cholesterol levels and are protective against NAFLD [88] possibly due to the loss-of-function variants in MARC1. 

TRIB1 is suggested to be involved in regulating hepatic glycogenesis and lipogenesis. shRNA knockdown or liver-specific deletion of *Trib1* in mice induced steatosis through increased glucose, cholesterol, FA, and triglyceride content [93,94].

Not much is understood about the functional relationship between the *ZF90* gene and NAFLD [60]. More is known about *CDH1,* which encodes E-cadherin, a tumour suppressor protein involved in cell–cell adhesion [60]. Liver-specific Cdh1 knockout mice fed a high-fat diet exhibit steatosis and spontaneously develop severe periportal and periductal inflammation and fibrosis compared to wild-type mice [95].

Whole-exome sequencing studies have also identified rare variants that could potentially be functionally associated with NAFLD [96,97]. The exome is the protein-coding region of the genome, and it is estimated that up to 60% of all known disease-causing variants are accounted for by exonic amino acid substitutions [98]. Genes associated with these exome-located variants include apolipoprotein B (*APOB*) (change at codon 2240 from lysine to a stop (p.K2240X)) and autophagy related 7 (*ATG7*) (rs143545741).

Similar to APOE, APOB also encodes for a component required in very-low-density and low-density lipoproteins for cholesterol transport [99]. *APOB* and its rare p.K2240X variant (stop gain) have both been associated with hepatic steatosis, and potentially with cirrhosis and liver cancer, according to longitudinal and large kindred-based studies [96,99]. However, the pathogenic impact of this particular *APOB* variant, and what pathways and mechanisms this variant affects remains unknown.

The rs143545741 *ATG7* (change at codon 426 from proline to leucine (p.P426L)) variant is hypothesised to be loss-of-function [97]. Individuals of European descent carrying this variant are at risk for severe NAFLD that is greater than seven-fold compared to the general population. The *ATG7* gene is involved in promoting autophagy via suppression of the autophagosome cargo protein p62 [97]. p62 is a known positive regulator of the NF-κB transcription factor that is central in the inflammatory pathway [100]. As such, the *ATG7* p.P426L variant could potentially cause unrestrained p62 expression, which may facilitate inflammation and hepatocellular ballooning [97]. 

### 1.7. Current and Potential Therapies

Characterisation of genetic variants is important for the development of therapeutic strategies. Current pharmacotherapies for NAFLD and NASH revolve around the general regulation of inflammatory cytokines, insulin resistance, and oxidative stress, because the pathogenesis of NAFLD and NASH is not well-understood, and the causes are multifactorial (environmental, metabolic, and genetic). As such, while these therapies may be significant for some individuals who have difficulty maintaining a healthy lifestyle, they may not prevent or alleviate the impacts of the genetic factors of NAFLD and may have adverse side effects [101].

Examples of existing pharmacological drugs include rosiglitazone and pioglitazone, which are thiazolidinedione insulin sensitizers that target the PPARγ signalling pathway. Individuals with NASH who were given either drug, in randomised controlled studies, showed improvements in serum aminotransferase levels and steatosis. However, no significant improvements in fibrosis were observed and both drugs induced adverse side effects (increased risks for ischemic heart disease and heart failure due to increased deposition of visceral fat) [38,101,102]. Obeticholic acid (OCA) is one of the more promising treatments for NAFLD and NASH, having reached phase 3 clinical trials [37]. Unlike rosiglitazone and pioglitazone, which do not interact with FXR [103], OCA is a selective farnesoid X receptor (FXR) agonist derived from chenodeoxycholic acid, a primary bile acid [36]. FXR is a highly expressed liver receptor (particularly in endothelial, hepatocytes and Kupffer cells) that has been shown, when activated, to reduce lipogenesis, inflammation, and liver fibrosis in individuals with NAFLD, NASH, or fibrosis and mice fed with a high-fat and high-carbohydrate diet [35,36,37]. 

Recently, in phase 2 clinical trials, using glucagon-like peptide-1 receptor (GLP-1R) agonists (like liraglutide and semaglutide) in patients with NAFLD has also been shown to significantly improve inflammation, hepatic steatosis, fibrosis, and promote weight loss [104]. By binding with its peptide hormone mimic, the GLP-1 receptor stimulates a glucose-regulatory response by modulating glucagon and insulin secretion. It is suggested that the response targets mechanisms involved in upregulating lipid oxidation and downregulating lipogenesis, resulting in increased FA removal, and reduces oxidative stress [104]. These mechanisms include increasing adiponectin and activating FXR. Investigating the effects of combined administration of OCA and a GLP-1R agonist (IP118) has been conducted only in mice, modelling NASH [105]. Co-administration of the drugs exhibited a greater (synergistic) effect on reducing ALT and AST, inflammation, steatosis, and fibrosis than OCA or IP118 only compared to the controls.

There is no doubt about the impact of genetics in NAFLD. As previously discussed, numerous GWAS and extensive functional studies have contributed to the *PNPLA3* p.I148M variant being well characterised as an effect modifier of NAFLD progression, and thus a potential therapeutic target. Currently, no approved pharmacotherapy exists for targeting this particular variant, which is associated with increased lipid accumulation, inflammation and fibrogenesis [106]. However, novel treatments developed in the future, with further study of the *PNPLA3* p.I148M variant, will hopefully alleviate a significant genetic risk factor of NAFLD and contribute to the advancement of personalised therapies. Even in the absence of specific therapeutic targeting of the *PNPLA3* gene, it may provide opportunities for personalised medicine, allowing important diagnostic and prognostic information that informs subsequent management and clinical prioritization.

## 2. Conclusions

NAFLD is a major cause of liver disease that affects a significantly increasing proportion of the global adult population. Improving outcomes relies on characterising its pathogenesis to better diagnose NAFLD, prognosticate, and inform pharmacotherapeutic strategies for personalised therapies. 

Environmental and metabolic disorders play prominent roles in NAFLD onset and progression to NASH and advanced liver disease. Yet, controlling for lifestyles and metabolic conditions suggests that other factors act as effect modifiers in the variability of NAFLD phenotype. This can partially be attributed to genetics. The interaction between genetics and the environment can explain the varying prevalence rates of NAFLD and NASH in different ethnic groups and populations who carry particular variants. These variants include the *PNPLA3* p.I148M, *TM6SF2* p.E167K, and *MBOAT7* rs641738 common variants. All these variants have been found to be associated with increased susceptibility to NAFLD and NASH, and functionally validated in in vitro and in vivo studies.

Numerous other gene variants associated with NAFLD and NASH have been identified through GWAS or whole-exome sequencing studies. The functional association of particular variants with NAFLD or NASH is still a work in progress. This includes, but is not limited to, such as those associated with the *APOE*, *GPAM*, *IL17RA*, *MARC1*, *TRIB1,* and *ZFP90-CDH1* genes, highlighted in this review. Determining the functional impact of the variant and the mechanistic pathogenic pathway remains critical. This is best highlighted with the *PNPLA3* p.I148M variant, which remains the strongest candidate gene in terms of effect size, yet remains poorly functionally characterised in terms of whether it is loss-of-function, gain-of-function, or neomorphic (novel function).

Currently, pharmacotherapies for NAFLD and NASH are focused on broadly regulating inflammatory cytokines, insulin resistance, and oxidative stress. This can be attributed to limited understanding about the pathogenesis and progression of NAFLD and NASH, as the disease is susceptible to multifactorial influences (environment, metabolic, and genetic). 

Significant progress has been made in understanding the genetics underlying NAFLD. Knowledge is emerging on the impact of genetic variation on NAFLD diagnosis, prognosis and treatment; however, much more work on the mechanisms is needed to allow therapeutic discovery and application of this knowledge, in order to address this burgeoning global problem.
